# A contamination-free and low-leakage-current Cu-TSV technology enabled by engineered double-sided processing

**DOI:** 10.1038/s41378-025-01018-x

**Published:** 2025-10-27

**Authors:** Yigang Hao, Yingtao Ding, Ziyue Zhang, Baoyan Yang, Jiaxuan Zhang, Huikai Xie, Zhiming Chen

**Affiliations:** School of Integrated Circuits and Electronics, Beijing Institute of Technology, 100081 Beijing, China

**Keywords:** Electrical and electronic engineering, Engineering

## Abstract

Through-silicon vias (TSVs) are crucial to the heterogeneous integration of multi-functional chiplets, providing high-performance vertical interconnects between different device layers. In this work, we introduce a novel TSV fabrication technology based on double-sided processing of polyimide-Ni (PI-Ni) functional layers, which can greatly simplify the TSV manufacturing flow, accompanying by advantages in improving TSV performance and reliability by effectively eliminating Cu contamination and significantly reducing leakage current. The key advancements of this approach include: (i) the complete coverage of the substrate with through-holes by the optimized double-sided PI insulation layer deposition process, where the chemical mechanical polishing (CMP) is carried out prior to the TSV metallization thus preventing substrate exposure to metallic contaminants; (ii) the deposition of a continuous Ni layer through an optimized double-sided electroless plating process, serving as both the barrier and the seed layer with high step coverage, enabling the simultaneous electroplating of TSV Cu conductors and double-sided redistribution layers (RDLs); and (iii) the demonstration of simulations and experimental validations highlighting improved thermo-mechanical stress distribution and superior electrical performance, including reduced parasitic capacitance and ultra-low leakage current. The engineered PI-Ni-based TSV fabrication technology offers a contamination-free Cu-interconnect solution for advanced post-Moore applications such as hetero-integrated microsystems containing IC chips and MEMS devices.

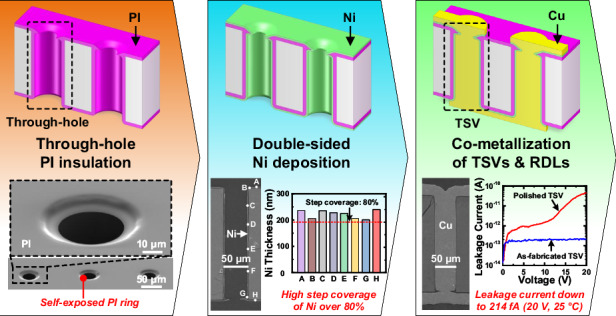

## Introduction

The rapid development of advanced technologies such as artificial intelligence (AI), high-performance computing (HPC), and Internet of things (IoT), has intensified the demand for miniaturized, high-density integration and packaging of diverse chiplets with multiple functionalities, materials, and processes. Based on through-silicon via (TSV) technology, 3D/2.5D integration has been proven effective for facilitating the heterogeneous assembly of multi-functional chips and devices within compact volumes^1,2^. In these configurations, TSVs serve as vertical electrical interconnects among different device layers, resulting in shorter interconnect lengths, improved signal transmission, and reduced system footprints compared to conventional interconnects like wire bonding^3,4^. However, the enhancement of the reliability and performance of TSVs is a prolonged but essential task, especially under the increasingly stringent demands of hetero-integrated systems such as efficient energy conversion. With the development of advanced electronic materials, it is promising to achieve functional and reliable TSV solutions, for example, a low-leakage-current and cost-effective TSV structure, by tailoring related processes.

**Fig. 1:**
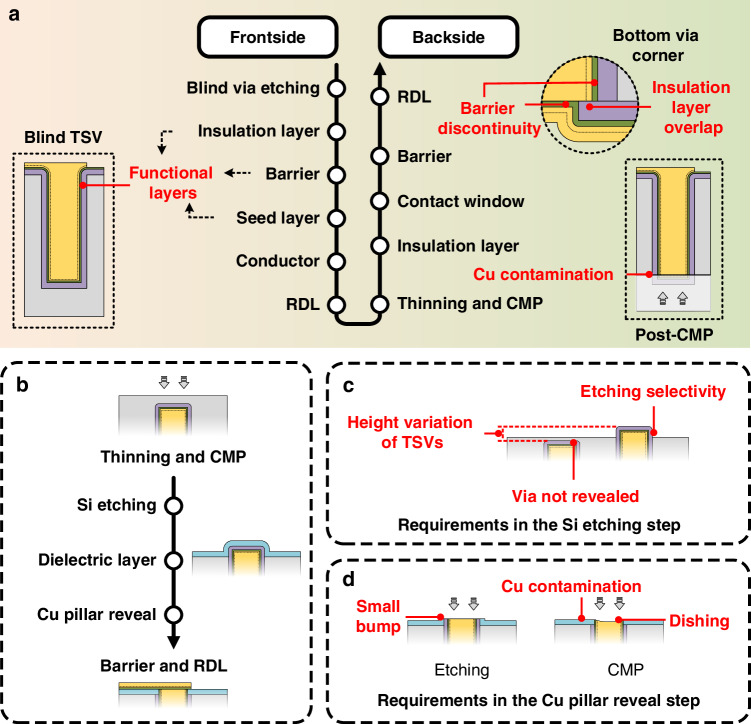
Schematics of typical TSV fabrication flows and related process concerns. **a** Traditional TSV fabrication flow. **b** Etch-back process. Requirements and risks of the etch-back process in **c** the Si etching step and **d** the Cu pillar reveal step

As shown in Fig. 1a, the traditional fabrication of TSVs is relatively complex, which typically begins with etching blind vias in Si substrates, followed by single-sided deposition of functional layers including the insulation layer, barrier, and seed layer. Cu electroplating is then carried out to fabricate the central conductors and accomplish the frontside planar metallization, and the redistribution layers (RDLs) are subsequently formed by patterning. After the frontside processing of blind TSVs, wafer thinning and chemical mechanical polishing (CMP) processes are performed on the backside of the substrate to expose the vias. However, the CMP process involves multiple materials including Cu, dielectric layer, and Si, which may cause Cu diffusion into the Si substrate, leading to larger leakage currents and compromised long-term reliability. Additionally, the Cu contamination can adversely affect the performance of adjacent electronic devices^5–7^. After CMP, the backside insulation layer is deposited and patterned for contact windows, which is desired to overlap part of the Cu conductor to ensure reliable connectivity with the sidewall insulation layer, breaking the barrier continuity, as illustrated in the upper right inset of Fig. 1a. Consequently, the discontinuity between the backside barrier and the sidewall barrier may also lead to Cu diffusion.

The etch-back process has emerged as a practical solution to mitigate Cu contamination, and a typical process flow is given in Fig. 1b. Initially, the backside CMP stops before exposing the vias. The subsequent TSV reveal generally consists of multiple steps including Si etching, dielectric layer deposition, and multi-material etching or CMP to expose the Cu pillars^7–10^. However, this process is complex with several passivation and removal steps. Besides, low TSV height variation is required to ensure high reveal yield during Si etching, alongside high etching selectivity to protect the sidewall insulation near the via bottoms, as illustrated in Fig. 1c. Furthermore, as shown in Fig. 1d, if etching is used for Cu pillar reveal, there could be small bumps influencing the surface flatness, while if CMP is used, it has to be precisely controlled with low polishing selectivity to avoid significant dishing. In addition, no matter whether the etch-back process is used, traditional TSV fabrication still involves subsequent backside processes for the deposition of the barrier and the RDLs, complicating the overall fabrication flow^11,12^.

The double-sided processing based on through-holes, which eliminates the need for backside TSV reveal steps, shows good potentials in achieving a more simplified TSV fabrication flow while simultaneously addressing the Cu contamination issue. Nevertheless, there are several challenges in the practical implementation of double-sided processes, especially the cost and yield concerns for industry. Conventional methods for insulation layer deposition, such as thermal oxidation of SiO_2_^13,14^ or laser drilling of polymer materials^15^, suffer from limitations due to high processing temperatures or the need for specialized equipment. Note that the use of polymer materials as alternatives to SiO_2_ for electrical insulation brings in advantages on both electrical performance and thermo-mechanical reliability^16,17^. Recently, a novel insulation layer fabrication method based on the spin-coating of polyimide (PI), a promising low-*k* polymer material for high-performance and energy-efficient electronic devices^18–20^, has been reported, which can deposit a polymer layer covering both the sidewalls of the through-holes and the double sides of the substrate^21^. As the PI has a lower dielectric constant than SiO_2_, this method can achieve lower parasitic capacitance of TSVs. However, considering that a ring structure is formed near the via opening during backside PI deposition, which may induce thermo-mechanical stresses and create difficulties for the subsequent barrier and seed layer deposition, more experiments and validations are required.

To achieve good continuity in barrier and seed layer fabrication, traditional physical vapor deposition (PVD) requires two rounds of sputtering from both sides of the sample^13,22^. While atomic layer deposition (ALD) is a reliable technique in depositing highly conformal layers^23,24^, its low deposition rates and high costs are of concern. Electroless plating, which is low-cost and low-temperature, has also been explored to create continuous Cu seed layers in through-holes^14^, yet certain additional processes such as sputtering are still necessary. Furthermore, some researchers have demonstrated the feasibility of using the electroless plated Ni as both the barrier and the seed layer in blind vias^25–27^, simplifying the TSV fabrication flow. Therefore, there is potential in simply utilizing electroless plating to realize the double-sided deposition of thin layers. However, this technique requires further exploration in through-holes to achieve the continuous co-deposition of barrier and seed layer for both the TSV sidewalls and the double-sided RDLs. As for the fabrication of the central Cu conductors in through-holes, both double-sided electroplating and single-sided bottom-up electroplating have been reported, while the former one is challenging to fully fill the through-holes without defects or voids^28^, and the latter one always requires an auxiliary metallized wafer as the initial growth point or an extra process to seal one side of the vias^28,29^. Therefore, it is significant to accomplish the fully-filled central Cu conductors in through-holes with a more simple and cost-effective method.

To satisfy key demands such as low leakage current and cost-effective processing for high-performance TSV interconnects, this study focuses on the development of double-sided processes engineered for employing PI-Ni functional layers in through-holes. We propose a novel TSV fabrication method where the backside reveal of the vias occurs during the deposition of the PI insulation layer, prior to the TSV metallization, thereby avoiding the Cu contamination issue. The fabrication processes for the PI-Ni functional layers, including the double-sided spin-coating and the double-sided electroless plating, are deeply investigated and technically optimized, achieving continuous Ni combined barrier and seed layer on the PI insulation layer. Moreover, electrical characterizations, particularly the comparison of the leakage current characteristics between the fabricated TSVs and those subjected to the multi-material backside CMP, demonstrate the effectiveness of the proposed fabrication method in avoiding the backside Cu contamination and obtaining TSVs with excellent electrical performance. In the following sections, the process details, surface characterizations, stress simulations, and electrical measurements will be presented and discussed.

## Methods

### Double-sided spin-coating of PI insulation layer

The PI insulation layer is fabricated based on the spin-coating of PI solution, which is mixed by PI and dimethylacetamide (DMAc) with an optimized volume ratio. Assisted by the vacuum treatment, a conformal PI layer is first deposited on the sidewalls of the blind vias and the frontside substrate surface. After curing, the frontside of the wafer is attached to a thermal tape to protect the frontside PI layer from contamination and then thinned from the backside until microns away from exposing the vias to avoid cracks and fractures, followed by a CMP process to expose the through-holes and planarize the substrate surface. Note that the CMP process primarily involves Si with a small portion of PI. After CMP, an ultrasonic cleaning step is carried out in acetone to remove the process-generated impurities inside the vias, such as the CMP abrasives and the polished Si and PI residues. Finally, the second spin-coating step of PI is carried out on the backside substrate surface with the frontside of the wafer attached to the thermal tape to enable the through-hole wafer to be fixed on the vacuum chuck of the spin-coater, where the film over the through-holes shrinks and exposes during the tailored stepped pre-curing procedure, forming a complete insulation layer together with the PI layer deposited from the frontside.

### Double-sided electroless plating of Ni combined barrier and seed layer

Off-the-shelf plating solutions are employed in this work for the electroless plating of Ni. This process comprises four steps: pretreatment, conditioning, adsorption of Pd ions, and reduction of Pd and Ni ions. To achieve similar deposition rates of Ni on both sides of the sample and facilitate the growth of Ni inside the vias, a new double-sided electroless plating technique is developed. The sample is suspended in the plating solutions by a clamp so that the reactions can take place on both sides. Besides, periodic flipping operations are employed, where the sample is flipped every 5 min during the 20-minute reduction step. In addition, the solutions are stirred to enhance the diffusion of the reactants and the removal of the generated bubbles.

### Simultaneous electroplating of Cu conductors and double-sided RDLs

Using the continuous Ni layer as both the barrier and the seed layer, Cu electroplating is conducted, enabling the simultaneous co-metallization of the Cu conductors of TSVs and the double-sided RDLs. Considering that there are PI rings at the backside via openings, the Cu electroplating process is carried out with the backside of the sample attached to the cathode plate. These ring structures actually facilitate the rapid sealing at the via bottoms in the superconformal growth of Cu, contributing to the complete filling of the vias and the simultaneous deposition of the Cu RDLs on both sides of the sample.

### Simulations and characterizations

Thermo-mechanical reliability of the TSV structure with PI ring is evaluated by simulating the von-Mises stress distribution using COMSOL Multiphysics, with results compared against standard TSV structures. Critical fabrication steps are characterized through top-view or cross-sectional scanning electron microscopy (SEM) observations. Surface roughness of the deposited PI insulation layer is measured using atomic force microscopy (AFM). Adhesion strength between the electroless plated Ni layer and the PI insulation layer is evaluated via standard cross cut tests according to the American Society of Testing Materials (ASTM) D3359-09. Additionally, elemental mapping and point analyses through the energy-dispersive X-ray spectroscopy (EDX) assess the continuity of the deposited Ni layer, as well as the potential Cu contamination on the Si substrate caused by the conventional multi-material CMP. Moreover, electrical characterizations of the fabricated TSVs on the DC resistance, parasitic capacitance, and leakage current characteristic are conducted using the Keysight B1500A semiconductor parameter analyzer with a Cascade Summit 11000 probe station.

## Results and discussion

### Engineered PI insulation layer

Achieving a continuous insulation layer across the TSV sidewall and substrate surface is crucial for maintaining the signal integrity. During the double-sided spin-coating of PI insulation layer, the exposure of the vias occurs at the CMP step, leading to some residual impurities inside the vias after CMP that are hard to be removed by directly cleaning with deionized (DI) water and rubber brush, as shown in Fig. S1a. Figure S1b presents the SEM images of the vias after the ultrasonic cleaning for 5 min with ultrasonic frequency and power of 28 kHz and 300 W, respectively. It can be seen that the impurities are removed and the sidewall PI is free of particles.

**Fig. 2:**
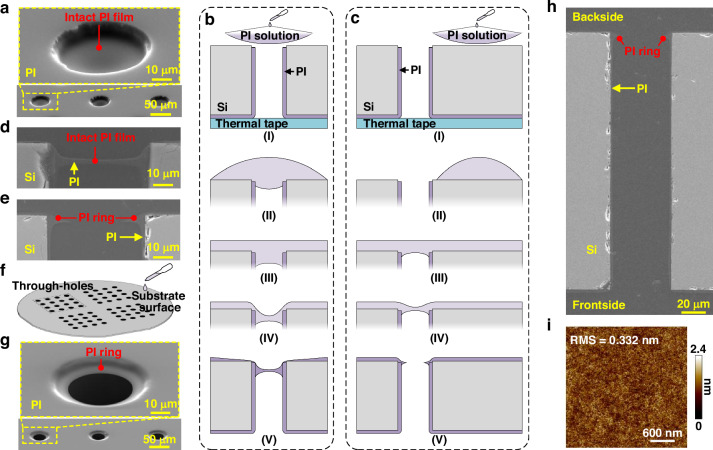
Schematics and fabrication results of the PI insulation layer deposition process. **a** Top-view SEM image of three failed structures with intact PI films hanging on the vias. Schematics showing the mechanisms of the backside PI insulation layer formation when the PI solution is dropped **b** directly onto the vias and **c** onto the substrate surface: (I) prior to PI dispensing, (II) during PI dispensing, (III) after PI dispensing, (IV) after spinning, and (V) after curing. **d**, **e** Cross-sectional SEM images of the fabricated backside PI insulation layers for the cases in (**b**, **c**), respectively. **f** Schematic of the suggested PI dispensing area. **g** Top-view SEM image of the fabricated backside PI insulation layer on a TSV array. **h** Cross-sectional SEM image of a complete PI insulation layer covering the entire substrate surface, including the through-hole. **i** AFM analysis showing the RMS of the backside PI insulation layer

In this work, the most challenging aspect of the PI insulation layer formation is the subsequent backside PI deposition step, which needs to form a PI layer on the backside substrate while exposing the via holes. The innovations of the engineered self-exposing backside PI deposition process can be explained from three aspects. First, key process parameters including the viscosity of PI and the spinning speed are carefully optimized to achieve a thin PI film over the via after spinning. Second, a novel pre-curing scheme is proposed, which is tailored by three stages with different curing temperatures, rather than the conventional one-step pre-curing at a constant temperature. As illustrated in Fig. S2, the solvent in the PI film starts to evaporate during the low-temperature stage. The film generally becomes thinner and finally breaks during the mid-temperature stage. In the last high-temperature stage, the self-exposed PI film with ring structure is pre-cured and shaped, after which it is fully polymerized with highest temperature of 240 °C. However, despite optimizing process parameters and employing a stepped pre-curing method, it is found that there might be a small quantity of failed structures exhibiting intact PI films without exposing the vias, as shown in Fig. 2a. Further observations indicate that the occurrence of these failures is associated with areas where the PI solution is initially dispensed onto the substrate. Therefore, the third optimization is proposed regarding the PI dispensing step.

Figure 2b, c illustrates the mechanisms of the backside PI insulation layer formation when the solution is dispensed on the via and the substrate surface, respectively. When the solution is dropped directly onto the via (Fig. 2bI), it naturally flows into the via due to gravity (Fig. 2bII). In contrast, when the solution is dispensed onto the substrate surface (Fig. 2cI), it spreads outward (Fig. 2cII) and covers the via (Fig. 2cIII). It is worth mentioning that the PI solution enters the via deeper for the case shown in Fig. 2bIII compared to Fig. 2cIII. After the spinning step, although the PI films hanging over the vias become thinner in both cases, the film in Fig. 2bIV is positioned deeper and thicker than the one in Fig. 2cIV, owing to the larger entrance depth of the PI solution inside the via prior to spinning. As a result, after the curing step, the PI film in Fig. 2cV breaks and exposes the via due to the shrinkage of the PI on the surrounding substrate surface, while the film in Fig. 2bV may remain intact.

The SEM images in Fig. 2d, e validate the above analyses, where there is a PI film hanging on the via at a relatively deeper position in Fig. 2d, while the PI layer in Fig. 2e successfully exposes and forms a ring structure near the via opening. Therefore, to avoid the issue caused by direct PI dropping, the PI solution should be dispensed on the substrate surface without vias, as illustrated in Fig. 2f. Figure 2g, h present the top-view and cross-sectional SEM images of the fabricated PI insulation layer in TSVs with a diameter of 50 μm, and the enlarged SEM images of the via bottom, via middle, and via top are shown in Fig. S3, respectively, showcasing that the proposed process can realize a continuous PI layer across the entire substrate surface including the sidewalls of the through-holes. The thicknesses of the frontside and backside PI layers are 2.3 μm and 1.5 μm, and the sidewall PI has a thickness ranging between 1.4 μm and 2.4 μm. Such continuous PI insulation layer with a relatively large thickness is beneficial for ensuring the electrical performance of the TSV structure. Moreover, it is noteworthy that the engineered self-exposing backside PI deposition process is applicable to TSVs with even smaller diameters to form PI rings considering the forming mechanism of the PI ring, which can be proven by the fabrication results presented in Fig. S4 where this process is implemented in TSVs with diameters of 18 μm and 11 μm, respectively. Besides, the surface roughness of the backside PI layer is measured by AFM, as shown in Fig. 2i. The measured root mean square (RMS) value from 65,536 points in a 3 μm × 3 μm area is only 0.332 nm, showcasing the good flatness. According to the SEM and AFM results, both the PI-Si interface and the PI surface are clean with no obvious impurities, ensuring the reliability of subsequent barrier and seed layer.

### Optimized double-sided Ni layer

**Fig. 3:**
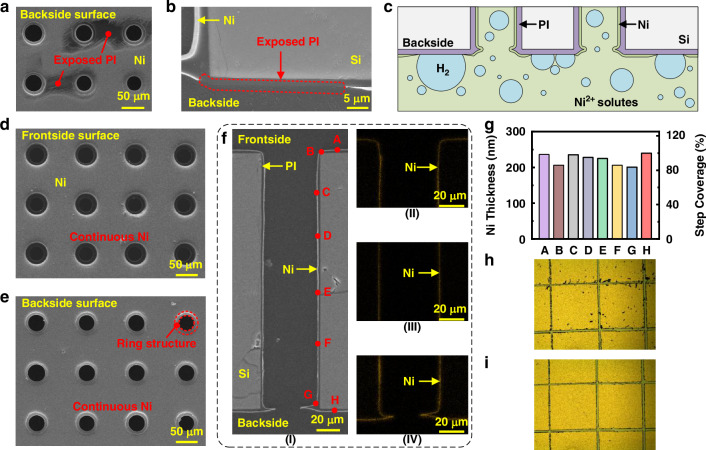
Fabrication results of the Ni electroless plating process. **a**, **b** Top-view and cross-sectional SEM images of the deposited Ni on the backside surface without flipping operations. **c** Schematic illustrating the cause of discontinuous Ni deposition. **d**, **e** Top-view SEM images of the Ni layer deposited on the frontside and backside surfaces with flipping operations, respectively. **f** Cross-sectional SEM image and EDX elemental mapping of the Ni layer deposited on the PI insulation layer covering the entire sample surface: (I) SEM image, (II)-(IV) EDX elemental mapping results at the via top, via middle, and via bottom, respectively. **g** Thickness and step coverage distributions of the Ni layer at typical positions of the TSV shown in (**f**). **h** Cross cut test result of the PI-Ni layers after scratching. **i** Cross cut test result of the PI-Ni layers after tape pressing and stripping

To achieve the double-sided deposition of Ni on the PI insulation layer, the sample is suspended in the plating solutions and periodically flipped during the electroless plating process. A control experiment without flipping operations is first carried out, where the backside of the suspended sample is downward in the solutions. Figure 3a, b shows the top-view and cross-sectional SEM images of the deposited Ni on the backside PI insulation layer after a 120-minute reduction step. It can be seen that the deposited Ni is not continuous, especially on the substrate surface near the via openings. This phenomenon can be explained as follows. During the electroless plating process, the Ni ions are reduced by hypophosphite (H_2_PO_2_^-^) with Pd nanoparticles as the catalysts, generating abundant H_2_ bubbles. As illustrated in Fig. 3c, the H_2_ bubbles are difficult to escape upwards, especially near the backside via opening, thus accumulating on the backside PI surface and obstructing the deposition of Ni.

With the introduction of the periodic flipping operations, the escape of the H_2_ bubbles from backside is significantly enhanced. The flipping operations facilitate bubble release in two ways: on the one hand, by mechanically vibrating the sample, the bubbles are easier to break or escape; on the other hand, by positioning the backside of the sample upward in the solution, the backside bubbles are allowed to rise and escape more easily. Figure 3d, e shows the frontside and backside surfaces of the sample after the Ni deposition process, respectively. It can be seen that the Ni layer is continuous on both sides, fully covering the PI insulation layer. Due to the PI ring structures stretching inwards as indicated in Fig. 3e, the exposed vias in Fig. 3e seem to be smaller than those in Fig. 3d. In addition, the duration of the Ni reduction step has been reduced to 20 min, highlighting another advantage of the optimized process. Figure 3f presents the cross-sectional SEM images of the Ni layer deposited on the sidewall of the through-hole, along with the EDX elemental mapping results of Ni at the via top, via middle, and via bottom as shown in Fig. S5. Figure S6 further demonstrates an enlarged SEM image of the via bottom containing a PI ring that is fully covered by a continuous Ni layer, which is obtained by direct hand-cleaving for a clearer observation on the morphologies of the PI and Ni layers. It can be concluded that the Ni layer is continuous across the entire sample surface including the PI ring, facilitating the subsequent co-metallization of the TSVs and the RDLs. Figure 3g plots the thickness and step coverage distributions of the deposited Ni layer at typical positions of the TSV in Fig. 3fI. The minimum step coverage exceeds 80%, indicating excellent conformality of the deposited Ni layer. Furthermore, Fig. 3h, i shows the cross cut test results of the adhesion strength between the PI-Ni functional layers. After scratching (Fig. 3h), there are some small Ni scraps near the edges of the grid patterns. However, as shown in Fig. 3i, these scraps are removed after the tape pressing and stripping, with the Ni grid patterns remaining intact and showing no peeling, demonstrating strong adhesion between the PI and Ni layers.

### Co-metallization of TSVs and RDLs

**Fig. 4:**
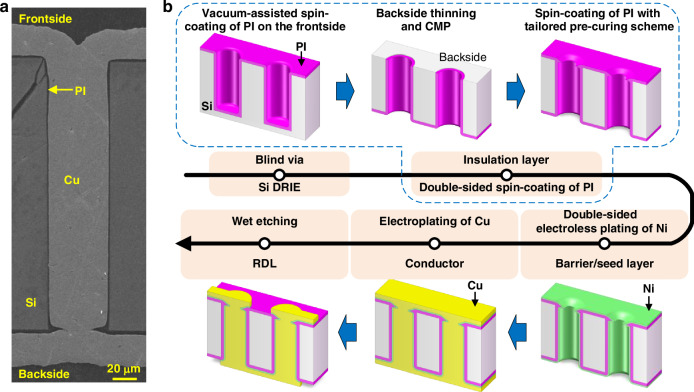
Fabrication results of the TSV structure and the proposed fabrication flow. **a** Cross-sectional SEM image of a fabricated TSV interconnect after Cu electroplating. **b** Schematics of the proposed double-sided fabrication flow for achieving contamination-free Cu-TSVs

Thanks to the continuous Ni seed layer across the entire sample surface, the co-metallization of the TSVs and the RDLs is achieved through the Cu electroplating process. The ring structures formed during the backside PI insulation layer deposition facilitate the rapid sealing of the via bottoms of the TSVs, after which the Cu conductors grow upward in a superconformal manner, effectively filling the vias and simultaneously depositing the RDLs on both sides of the sample. Figure 4a shows a fabricated TSV structure after electroplating, with a TSV diameter of 50 μm and height of 220 μm. It can be seen that the Cu conductor is intact and dense both inside the via and on the double-sided surfaces, without voids or cracks, confirming the high quality of the seed layer and the feasibility of the fabrication process.

In summary, a novel fabrication method based on double-sided processing of the PI-Ni functional layers is proposed in this work, with the schematic flow shown in Fig. 4b. First, blind vias are created through deep reactive ion etching (DRIE). A continuous PI insulation layer is then deposited using the tailored double-sided spin-coating method, which involves three steps including the vacuum-assisted spin-coating of PI in the blind vias, backside thinning, and CMP to expose the vias, and spin-coating of PI on the backside surface featuring the novel pre-curing scheme, as illustrated in the inset of Fig. 4b. Notably, the CMP process is performed prior to the metallization steps, preventing Cu contamination on the Si substrate. Next, a uniform Ni layer, serving as both the barrier and the seed layer, is deposited on the PI insulation layer, covering the via sidewalls and both sides of the sample. This process utilizes the optimized double-sided electroless plating technique with periodic flipping operations, enabling the co-metallization of the TSVs and the RDLs. The co-metallization is then achieved through Cu electroplating, during which the Cu conductors and the double-sided RDLs are deposited simultaneously. Finally, the RDLs are patterned by lithography and wet etching. Note that the thickness of the fabricated RDLs is relatively large due to the sufficient electroplating duration to fully fill the vias, there are some optional solutions to obtain a thinner RDL thickness if required, for example, conducting a carefully controlled wet etching on the whole substrate surface without masks or an additional Cu CMP to uniformly decrease the Cu thickness.

### Thermo-mechanical stress distribution simulations

There have been some works of literature investigating and verifying the thermal reliability of TSVs with polymer insulation layers including PI in terms of von-Mises stress distribution^30–33^, thermal stability^34–36^, annealing-induced protrusion^37,38^, et al. For example, some previous studies have demonstrated that using PI as the insulation layer material can mitigate the von-Mises stresses in the substrate surface near the TSVs, compared to traditional SiO_2_ insulation layers^30–33^. However, the ring structure at the via bottom may raise concerns regarding the stress distribution characteristic of the proposed TSV structure, which is a potential risk that could be caused by the ring structure with a tip-like edge. To address this, we conduct a series of finite element analysis (FEA) simulations to validate the thermo-mechanical reliability of the proposed TSV structure.

**Fig. 5:**
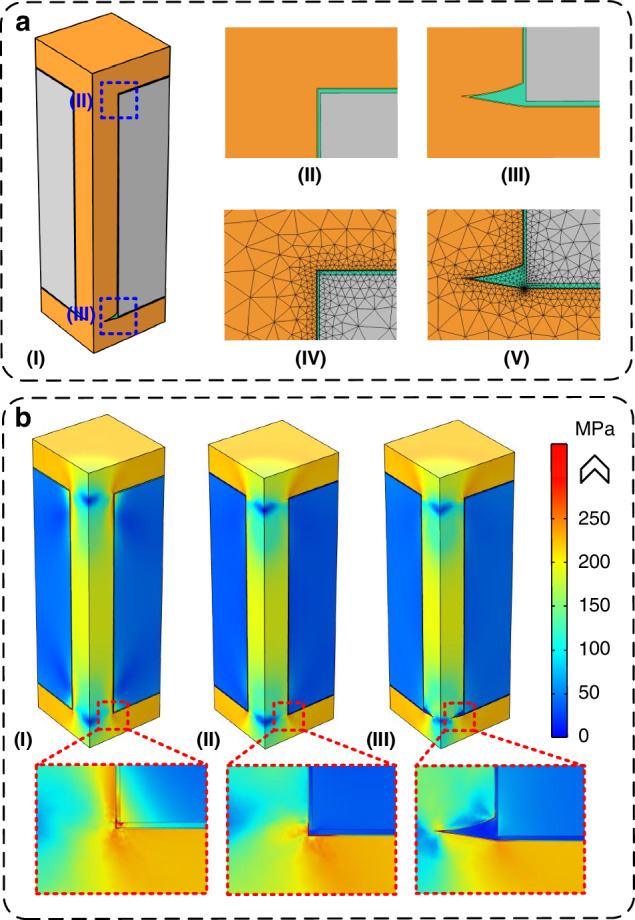
Simulation results of the thermo-mechanical stress distribution. **a** Configurations and meshing of the FEA model: (I) basic model, (II) via corner without ring structure, (III) tailored via corner with ring structure, (IV) and (V) meshing of the regions near the via corners without and with ring structure, respectively. **b** Simulated stress distributions for TSVs with (I) SiO_2_ insulation layer, (II) PI insulation layer without ring structure, and (III) PI insulation layer with ring structure

Three different configurations are considered in the simulations: the first case uses SiO_2_ as the insulation layer without any ring structure, the second case employs PI insulation layer without PI ring, and the third case is designed according to the fabricated structure with PI ring to assess the effects of the ring, and its FEA model is shown in Fig. 5aI. The via corners of the FEA models, without and with the ring structure, are illustrated in Fig. 5aII, III, respectively. Figure 5aIV, V shows the corresponding meshing of these critical regions. For the simulation method, an advanced element birth and death technique is employed to take the residue stresses introduced by the various processing steps into consideration^30,37,39^. The reference temperatures are set according to the practical fabrication processes, and Cu is modeled as an elastic-plastic material, while the other materials are assumed to be isotropic and linearly elastic^37,40^.

Figure 5b shows the simulation results of the thermo-mechanical stress distributions of the three configurations. Three important conclusions can be inferred from the results. First, replacing the traditional SiO_2_ insulation layer with PI reduces the von-Mises stresses in both the TSV structure and the surrounding substrate surface, as shown in Fig. 5bI, II. Second, as illustrated in the insets of Fig. 5bII, III, the presence of PI rings does increase the local stress, particularly at the edge of the ring. This is primarily due to the continuous yet thin Ni layer covering the ring, which is relatively sharp at the edge. Third, although the maximum stress increases in the ring region, it remains comparable to or even lower than that in the SiO_2_ case, indicating that the structure is still feasible. Notably, most areas of the model in Fig. 5bIII exhibit stress distributions similar to those in Fig. 5bII, with the exception of the ring region. Figure S7 compares the stress distributions on the backside substrate surfaces for the three cases, where the extraction path starts from the via edge and goes outwards in the radial direction, as illustrated in the inset. Therefore, the benefits of using PI as the insulation layer material are still effective, and the devices near the TSVs will experience less stress compared to those with SiO_2_ insulation layers, despite such issue is actually less influential for the deep TSVs in hetero-integrated microsystems containing IC chips and MEMS devices compared to the fine-diameter TSVs in high-density 3D-ICs such as memory stacks, as the requirements on the TSV density and pitch are less significant than that on the TSV depth in thick interposers.

### Electrical characterizations

**Fig. 6:**
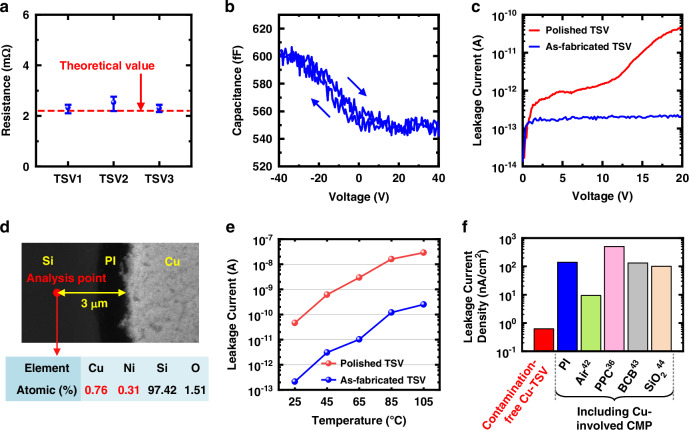
Electrical measurement results. **a** DC resistance values for three different TSVs. **b** C-V curve for a single TSV. **c** Leakage current characteristics for an as-fabricated contamination-free Cu-TSV (blue line) and a polished TSV (red line). **d** SEM image of a local area on the backside of the polished sample, with EDX point analysis showing elemental composition at a specific location 3 μm away from the central Cu conductor. **e** Comparison of leakage current values at various temperatures under a 20 V bias between the as-fabricated contamination-free Cu-TSV (blue line) and the polished TSV (red line). **f** Comparison of leakage current density of the as-fabricated contamination-free Cu-TSV (in red) and polished TSV (in blue) in this work and other reported TSVs with various insulation layer materials

To evaluate the electrical performance of the fabricated TSV interconnects, a series of electrical measurements is conducted. First, the DC resistance of a single TSV interconnect is measured utilizing a Kelvin structure, as depicted in Fig. S8. The structure includes three TSVs connected by backside RDLs, and the central one is the device under test (DUT). During the measurement, a constant voltage of 100 mV is applied to the left two TSVs, and the current flowing through the DUT is recorded. Simultaneously, a small constant current of 10 μA is applied to the right two TSVs, and the voltage between these TSVs is measured in real time. Since the current in the right TSV is tiny, the measured voltage value can be treated as the voltage drop between the top and bottom of the DUT. Therefore, its resistance is calculated by dividing the measured voltage by the measured current from the left two TSVs. Multiple measurements are carried out on three TSVs located at different positions on the sample, all with the same dimensions as the TSV shown in Fig. 4a. As shown in Fig. 6a, the average resistance value of a single TSV, obtained from 100-time repeated measurements, ranges between 2.30 mΩ and 2.53 mΩ, which is close to the theoretical value^41^.

Next, the parasitic capacitance between the TSV and the Si substrate is evaluated by sweeping the voltage bias from −40 V to 40 V and then back to −40 V, and the measured capacitance-voltage (C-V) curve is given in Fig. 6b. It can be seen that the TSV exhibits a small accumulation capacitance of approximately 603 fF and an even smaller depletion capacitance of 542 fF. These low parasitic capacitances are primarily attributed to the PI insulation layer, which has a low dielectric constant, and contribute to improving the electrical performance of the TSV interconnects, including reduced signal latency, crosstalk, and power loss. A small clockwise hysteresis exists between the forward and reverse bias cycles, which can be mainly attributed to the interface states and traps near the PI-Si interface. Note that the capacitance is relatively stable with a tiny variation below 20 fF within the typical operating voltage of interests ( ~ 0 − 5 V), contributing to enhancing the stability of the integrated system.

To verify the enhancement in electrical performance offered by the proposed contamination-free Cu-TSVs, another sample is prepared, where the TSVs are polished from the backside after fabrication to expose the Si substrate, referred as “polished TSVs”. This sample imitates the traditional TSV fabrication process that suffers from possible Cu contamination due to the backside multi-material CMP. After CMP, the surface is cleaned by a specialized acidic solution to remove the metal particles adsorbed on the surface that are generated during processing. The leakage current characteristics of the polished TSVs are then measured and compared to those of the TSVs fabricated via the proposed fabrication flow in this work.

Figure 6c plots the measured leakage currents of an as-fabricated TSV and a polished TSV obtained at room temperature. As shown by the blue line in Fig. 6c, the leakage current from the as-fabricated TSV to the Si substrate is only 214 fA at 20 V, with a leakage current density of 0.619 nA/cm^2^. This ultra-low leakage current can be attributed to the excellent insulating properties of the continuous and thick PI insulation layer, free from Cu contamination, and the effective barrier characteristic provided by the continuous Ni layer. In contrast, after the backside CMP process, the leakage current increases by two orders of magnitude to 46 pA at 20 V, as shown by the red line in Fig. 6c. This deterioration is due to the multi-material CMP process, which may spread polished Cu particles to the insulation layer and the substrate, causing contamination. Figure 6d presents the EDX point analysis results of an analysis point which is 3 μm away from the central Cu conductor of the polished TSV sample, as indicated in the SEM image. The detected atomic proportions of Cu and Ni elements are 0.76% and 0.31%, respectively, which are introduced by the backside CMP from the TSV region. Figure S9 further shows the detected atomic proportions of Cu and Ni elements from a series of analysis points across the PI insulation layer, revealing the trends of the metal materials to be smeared out towards the substrate. Therefore, although an acid cleaning step is used to remove the metal particles, there are still some stubborn particles that have been tightly attached on the surface or penetrated into the substrate. These residuals are tough to be fully removed and form contaminants influencing the device performance.

Furthermore, the leakage current characteristics of both the contamination-free Cu-TSVs and the polished TSVs are measured under a range of temperatures. As shown in Fig. S10, the leakage current of the contamination-free Cu-TSV increases with temperature, rising from 214 fA at room temperature to 248 pA at 105 °C under a 20 V bias. Figure 6e compares the leakage current values of the contamination-free Cu-TSV with those of the polished TSV at various temperatures. It can be seen that the TSV on the polished sample exhibits significantly worse electrical performance and thermal reliability, with leakage current values approximately two orders of magnitude higher than those of the contamination-free Cu-TSV. For instance, the leakage current reaches 28.6 nA at 105 °C in the polished sample.

Figure 6f further presents the comparison of leakage current density of the contamination-free Cu-TSV and polished TSV in this work and several reported TSVs in literature with insulation layer materials including air^42^, polypropylene carbonate (PPC)^36^, benzocyclobutene (BCB)^43^, and SiO_2_^44^. Note that the TSVs given in Fig. 6f experience Cu-involved CMP except for the contamination-free Cu-TSV. These measurement and comparison results clearly demonstrate the effectiveness of the proposed fabrication method for manufacturing contamination-free Cu-TSVs, which exhibit superior electrical performance, particularly in terms of ultra-low leakage current. This improvement is crucial for the high-performance hetero-integrated microsystems containing IC chips and MEMS devices.

## Conclusions

In this work, a novel TSV fabrication technology is demonstrated, where the backside CMP step is carried out after the sidewall PI layer deposition, prior to the backside PI deposition and metallization, and the co-metallization of the TSVs and the RDLs is achieved simultaneously using double-sided processing techniques. By engineering the double-sided processing of PI-Ni functional layers based on through-holes, contamination-free Cu-TSV interconnects are successfully fabricated with simplified and cost-effective processes. Thermo-mechanical FEA simulations confirm that the introduction of the PI ring does not compromise the reliability of the proposed TSV structure. In fact, the maximum von-Mises stress in the new structure is lower than that in the traditional TSV with SiO_2_ insulation layer. Furthermore, the fabricated TSVs demonstrate excellent electrical performance, with an ultra-low leakage current of only 214 fA at 20 V at room temperature. The leakage current is two orders of magnitude smaller than that of the TSVs undergoing the backside multi-material CMP process. These findings validate the feasibility and practicability of the proposed fabrication technology for manufacturing high-performance TSV interconnects, showing great potentials for the heterogeneous integration of multi-functional chiplets in the post-Moore era.

## Supplementary information

Final SI_clean
