# Supplementary material for: A contamination-free and low-leakage-current Cu-TSV technology enabled by engineered double-sided processing
Source: Microsyst Nanoeng. 2025 Oct 27;11:199. doi: 10.1038/s41378-025-01018-x (PMC12559307; doi:10.1038/s41378-025-01018-x)
Supplement: Supplementary file 1 — Final SI_clean [file 41378_2025_1018_MOESM1_ESM.doc]

**Supplementary Information**

**A contamination-free and low-leakage-current Cu-TSV technology enabled by engineered double-sided processing**

**Yigang Hao, Yingtao Ding, Ziyue Zhang^*^, Baoyan Yang, Jiaxuan Zhang, Huikai Xie, and Zhiming Chen^*^**

School of Integrated Circuits and Electronics, Beijing Institute of Technology, Beijing 100081, China.

*zyzhang@bit.edu.cn; czm@bit.edu.cn


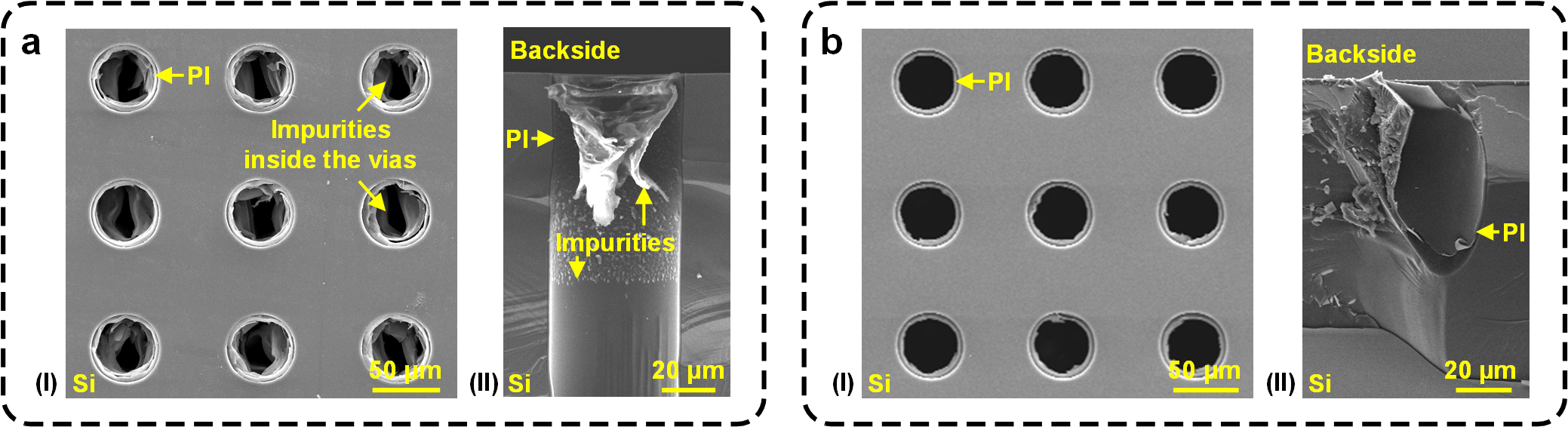


**Fig. S1** **SEM images of the vias after CMP. a** (I) Top-view and (II) cross-sectional images of the vias before the ultrasonic cleaning. **b** (I) Top-view and (II) cross-sectional images of the vias after the ultrasonic cleaning. There are some impurities inside the vias after CMP, while they are completely removed after the ultrasonic cleaning. The abnormal cracks and particles around the vias in the cross-sectional images can be attributed to the hand-cleaving operations to observe the sidewall PI layers more directly.


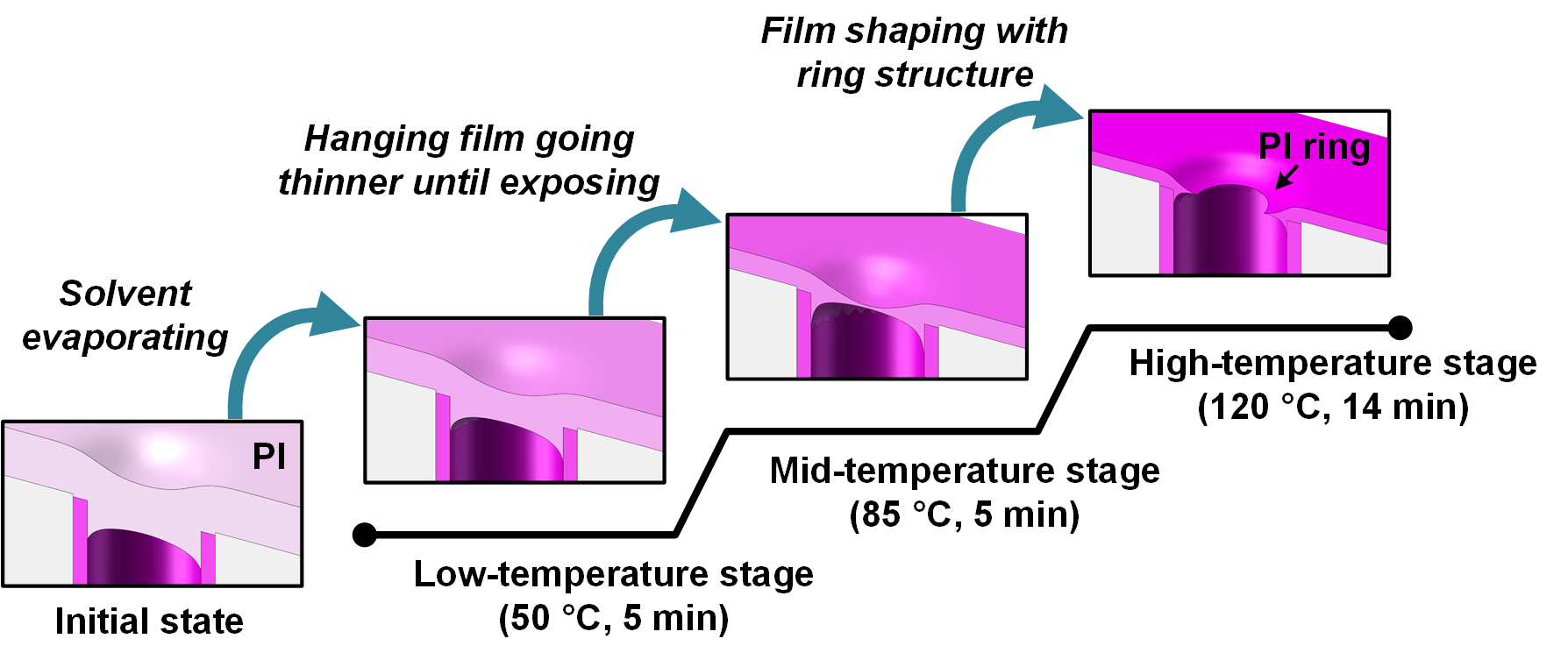


**Fig. S2** **Schematic of the tailored pre-curing step composed of three stages with different curing temperatures.** The solvent in the PI film starts to evaporate during the low-temperature stage (50 °C, 5 min). The film continues to become thinner during the mid-temperature stage (85 °C, 5 min), during which it finally breaks. The third high-temperature stage shapes the self-exposed PI film with ring structure. The PI film is fully polymerized with highest temperature of 240 °C.


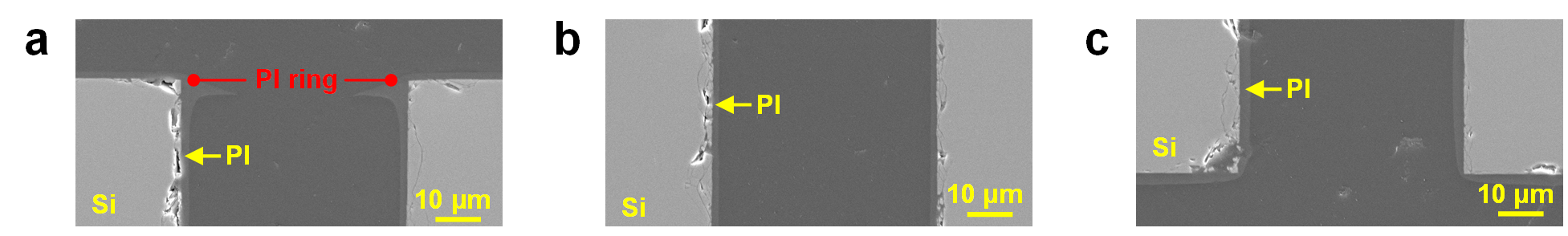


**Fig. S3** **Enlarged cross-sectional SEM images of the fabricated PI insulation layer in Fig. 2h. a** Via bottom. **b** Via middle. **c** Via top.


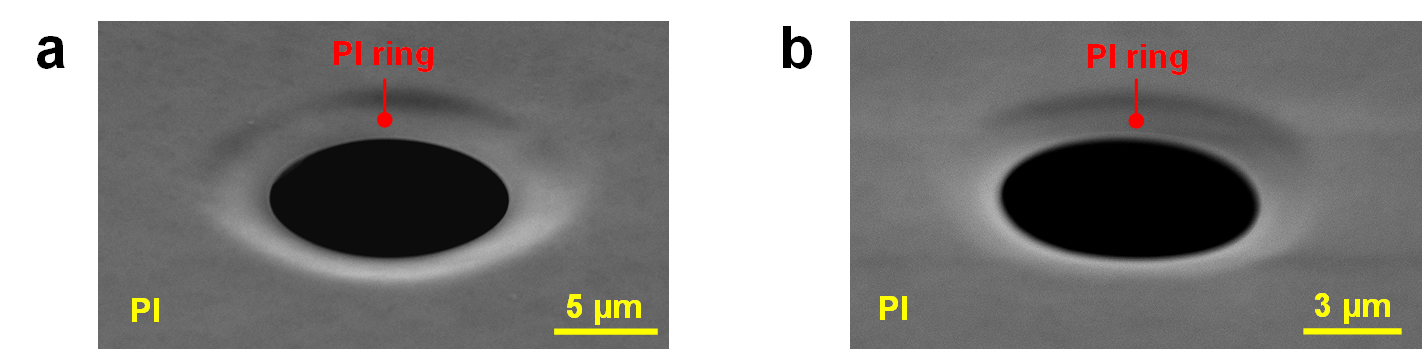


**Fig. S4** **Enlarged top-view SEM images of the fabricated PI ring structures in TSVs with smaller diameters. a** 18 μm, **b** 11 μm.


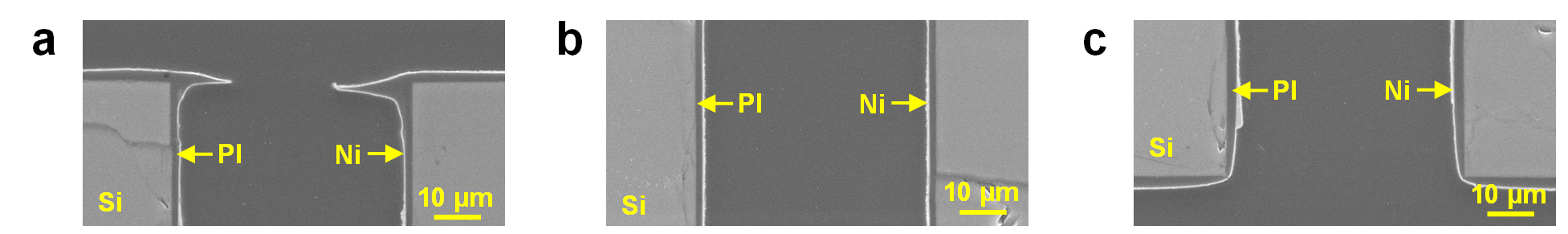


**Fig. S5** **Enlarged cross-sectional SEM images of the fabricated Ni layer in Fig. 3f(I). a** Via bottom. **b** Via middle. **c** Via top.


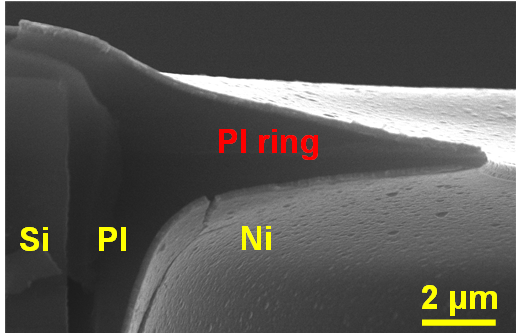


**Fig. S6** **Enlarged cross-sectional SEM image of a via bottom obtained by hand-cleaving before Cu electroplating.** The PI ring is fully covered by the continuous Ni layer.


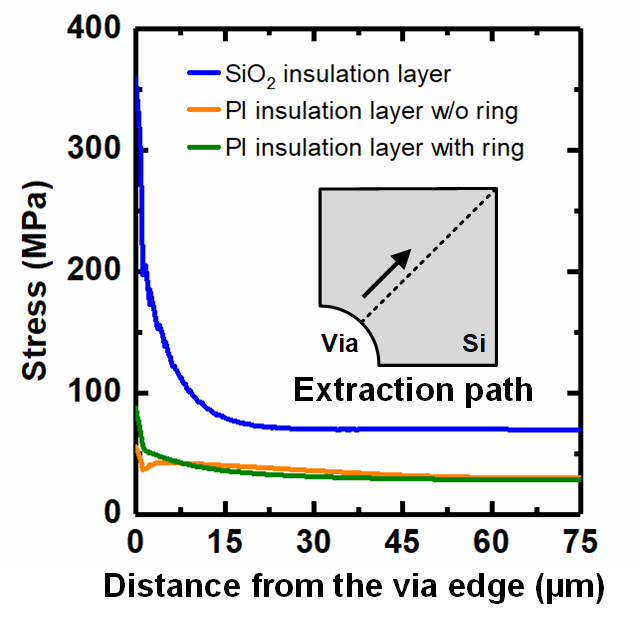


**Fig. S7** **Extracted thermo-mechanical stress distributions on the backside substrate surfaces for the three TSV configurations**: SiO_2_ insulation layer (blue line), PI insulation layer without ring structure (orange line), and PI insulation layer with ring structure (green line). The inset shows the extraction path starting from the via edge.


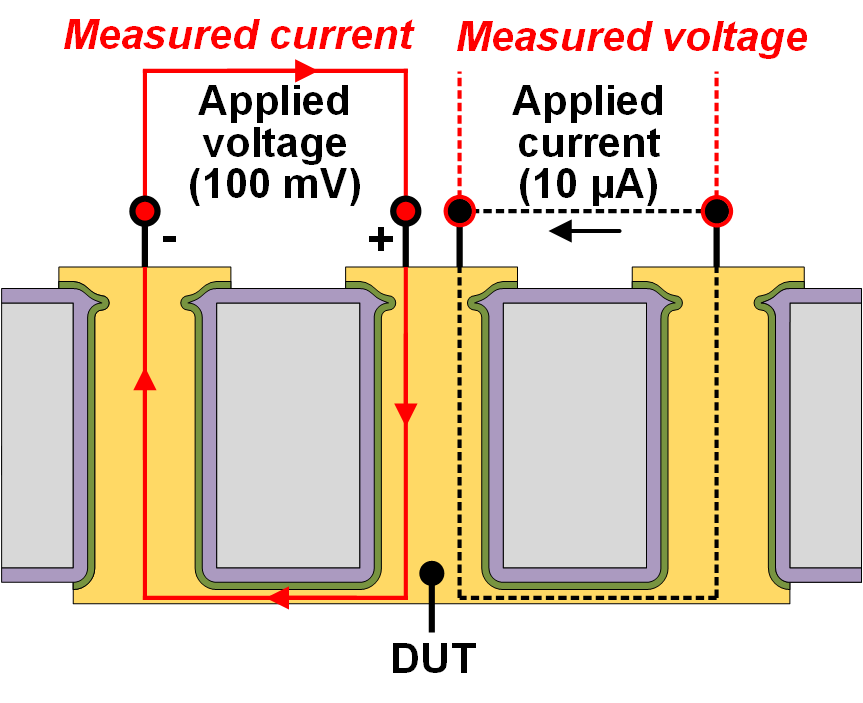


**Fig. S8 Schematic of the Kelvin structure for DC resistance assessment.** A constant voltage of 100 mV is applied to the left two TSVs, and a constant current of 10 μA is applied to the right two TSVs. The current flowing through the left two TSVs and the voltage between the right two TSVs are measured. Then the resistance of the DUT is calculated by dividing the measured voltage by the measured current.


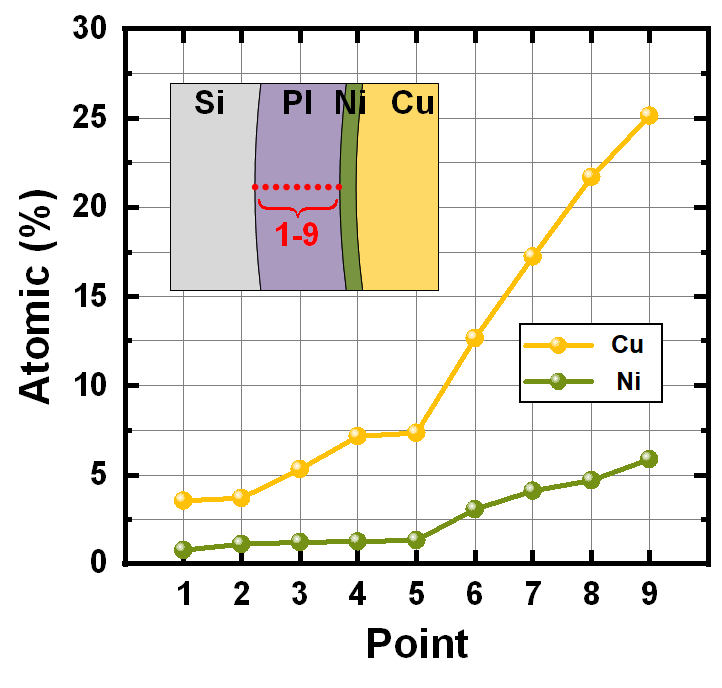


**Fig. S9** **Detected atomic proportions of Cu and Ni elements by EDX point analyses from a series of analysis points on the PI insulation layer.**


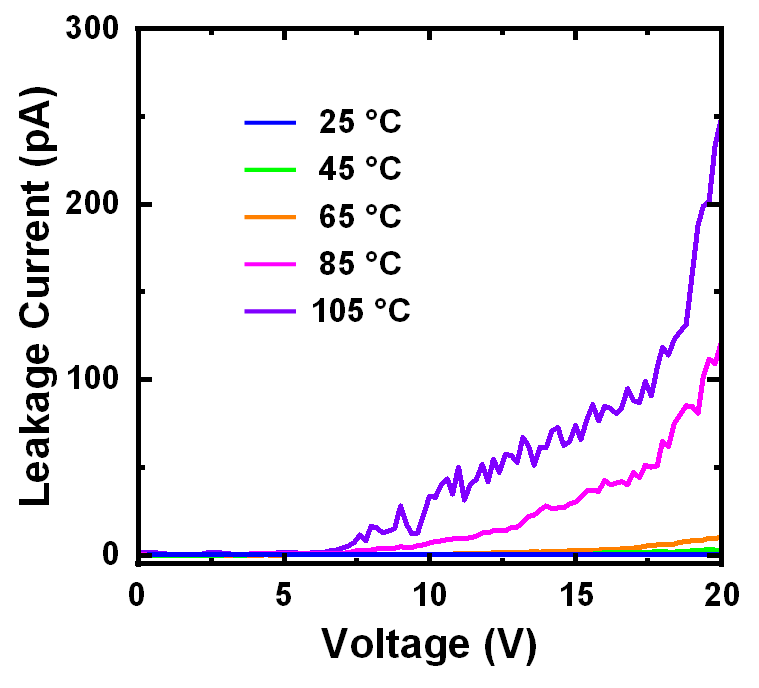


**Fig.** **S10** **Measured leakage current characteristics for a contamination-free Cu-TSV at various temperatures.**
